# Animal Studies on the Effects of Edible Bird’s Nest on Cognitive Function and Neuroprotection: A Systematic Review

**DOI:** 10.3390/nu18091373

**Published:** 2026-04-27

**Authors:** Jiaying Chi, Yu Shan Tan, Hemaniswarri Dewi Dewadas, Chai Nien Foo, Yang Mooi Lim

**Affiliations:** 1 Centre for Cancer Research, M. Kandiah Faculty of Medicine and Health Sciences, Universiti Tunku Abdul Rahman, Kajang 43000, Selangor, Malaysia; 17835207335@163.com (J.C.); foocn@utar.edu.my (C.N.F.); 2 Centre for Biomedical and Nutrition Research, Faculty of Science, Universiti Tunku Abdul Rahman, Kampar 31900, Perak, Malaysia; hemaniswarri@utar.edu.my; 3 Department of Business and Public Administration, Teh Hong Piow Faculty of Business and Finance, Universiti Tunku Abdul Rahman, Kampar 31900, Perak, Malaysia; 4 Department of Population Medicine, M. Kandiah Faculty of Medicine and Health Sciences, Universiti Tunku Abdul Rahman, Kajang 43000, Selangor, Malaysia; 5 Department of Pre-Clinical Sciences, M. Kandiah Faculty of Medicine and Health Sciences, Universiti Tunku Abdul Rahman, Kajang 43000, Selangor, Malaysia

**Keywords:** edible bird’s nest, EBN, cognitive function, neuroprotection, animal studies

## Abstract

Objectives: This systematic review aims to evaluate the effects of Edible Bird’s Nest (EBN) extract on cognitive function and neuroprotection in animal models and systematically review the relevant research evidence. Methods: A systematic search was conducted in the databases of PubMed, Scopus, Web of Science, EMBASE, Taylor Francis, Wiley, and Cochrane Library for relevant research published up to October 2025. Search terms included “Edible Bird’s Nest”, “Bird’s Nest Extract”, “EBN”, “Swiftlet nest”, “Collocalia”, “Cognitive”, “Memory”, “Learning”, “Neuroprotection”, “Brain”, “Neural”, “Neurotrophic”, “Animal”, “Mice”, “Mouse”, “Rat”, “Rats”, “In vivo”, and “Model”. Two researchers independently screened all the relevant articles, extracted relevant data, and assessed the quality of included studies using the Systematic Review Centre for Laboratory Animal Experimentation (SYRCLE) risk of bias assessment tool. Results: This systematic review included 11 animal studies, primarily focused on rodent models. Preclinical evidence suggests that Edible Bird’s Nest extract (EBN) may improve performance in several cognitive function tests. Animals treated with EBN appeared to show enhanced spatial memory and learning abilities in experimental settings. At the molecular level, the EBN treatment group showed improved antioxidant capacity and reduced neuroinflammation. Additionally, EBN promoted the expression of neuroprotective factors and enhanced synaptic plasticity. Research suggests that appropriate doses of EBN may have beneficial effects on cognitive enhancement and can alleviate cognitive dysfunction and neuropathological changes. Conclusions: Preliminary evidence from this systematic review suggests that EBN appears to show protective and potentially enhancing effects on cognitive function in animal models. EBN works through multiple mechanisms, including antioxidant and anti-inflammatory effects, as well as promoting the expression of neurotrophic factors and synaptic plasticity. These findings provide initial support for further investigation of EBN as a potential neuroprotective agent and cognitive enhancer. However, there is heterogeneity and methodological limitations in the research, and more standardized studies and preclinical translational research are needed to further validate the application potential of EBN in neuroprotection. These results provide an important reference for developing EBN-based functional foods and supplements for the prevention and adjuvant treatment of cognitive impairment and neurodegenerative diseases.

## 1. Introduction

Edible Bird’s Nest (EBN) is a secretion produced by swiftlets [1], which is constructed using their saliva mixed with feathers, grass leaves, or plant stems in coastal caves along the cliffs of Southeast Asia and is abandoned after the young swiftlets hatch and mature. Since the Tang Dynasty (618–907 AD) and the Song Dynasty (960–1279 AD), people have enjoyed this delicacy. EBN is sold at very high prices due to its high nutritional and medicinal value, making it one of the most expensive animal products in the world. It is commonly referred to as the “Caviar of the East” [2].

EBNs are known for their potential effects, attributed to their unique nutritional composition and properties. EBN consists of high-value glycoproteins and is rich in amino acids, carbohydrates, calcium, sodium, and potassium [3]. These components not only promote cell repair and tissue regeneration but also exhibit remarkable effects in enhancing immunity, delaying aging, and improving skin health [4]. Additionally, the bioactive components of EBN are believed to help regulate metabolism and support cardiovascular health, and are used in traditional Chinese medicine as a superior tonic [5]. In addition, the efficacy and medical value of EBN are recorded in more than 20 classics of Chinese medicine. According to the Essential of Materia Medica (*Ben Cao Bei Yao*), EBNs nourish the lungs, dissolve phlegm, relieve cough, and provide gentle nourishment, making them an excellent remedy for combating fatigue [6]. Due to its wide-ranging benefits, EBN has become one of the most sought-after luxury products worldwide.

Modern scientific research has further revealed the potential health benefits of EBN, particularly in the areas of neuroprotection and cognitive enhancement [7]. EBN is rich in sialic acid, which constitutes approximately 11% of its composition [8]. This unique abundance of sialic acid plays a crucial role in promoting brain cell growth, enhancing synaptic transmission, and supporting brain development [9], while also contributing to improved cognitive function, strengthened immunity, and cell proliferation. Furthermore, EBN possesses significant antioxidant and anti-inflammatory properties, effectively reducing oxidative stress caused by free radicals and safeguarding nerve cells from damage [10]. These characteristics show potential not only in preventing neurodegenerative diseases but also in improving overall brain health. As a nutrient-rich natural food, the health benefits of EBN have been validated by modern science, providing a solid foundation for its development as a functional food [11].

Neurodegenerative diseases are among the most severe health issues affecting millions of people worldwide, with their incidence rising sharply as life expectancy increases. Their prominent characteristic is the loss of neurons [12]. Among these diseases, Alzheimer’s disease (AD) and Parkinson’s disease (PD) are relatively common, while Huntington’s disease (HD), amyotrophic lateral sclerosis (ALS), frontotemporal dementia (FTD), corticobasal syndrome (CBS), multiple system atrophy (MSA), and progressive supranuclear palsy (PSP) are comparatively rare [13]. The causes of these diseases may be linked to genetic factors, environmental influences, or complex pathological processes. Many neurodegenerative diseases may eventually progress to dementia. It is estimated that by 2050, the global number of dementia patients will approach 150 million, resulting in an economic burden of approximately 10 trillion USD and imposing profound psychological and social impacts on both patients and their families [14].

Although there has been some progress in understanding the pathological mechanisms of neurodegenerative diseases, treatment strategies remain inadequate [15]. Current therapies primarily focus on symptom management and fail to address the root cause of neuronal loss [16], making the development of effective drugs extremely challenging [17]. Furthermore, due to the lack of disease-modifying treatments for neurodegenerative diseases, patients must rely on daily care to cope with their gradual health deterioration [18].

Against this backdrop, natural products, with their unique biological activities, show great potential in neuroprotection; integrating natural products into treatment strategies may offer a multifaceted approach to mitigating the progression and burden of neurodegenerative diseases, ultimately improving patient outcomes [19].

Studies have shown that EBN has potential neuroprotective effects, but its precise molecular mechanisms remain unclear and require further investigation [20]. Moreover, significant variations in experimental designs across existing studies make it difficult to compare results and highlight the lack of a standardized research framework. Most studies have focused on short-term effects, while the exploration of EBN’s long-term efficacy and safety remains limited, preventing a comprehensive evaluation of its sustained neuroprotective impact. More importantly, there is a lack of disease-specific research, as well as translational studies bridging the gap between animal experiments and human clinical applications, making it challenging to effectively utilize findings from animal models for the development of therapeutic strategies for humans.

The rich bioactive components of EBN offer the potential for developing novel, multifaceted neuroprotective strategies. Future research could focus on the development of personalized treatments targeting specific neurodegenerative diseases, with efficacy validated using relevant animal models [21]. Additionally, establishing standardized preparation methods for EBN extracts would ensure the reproducibility and consistency of research outcomes [22], thereby laying a solid foundation for further clinical applications.

As shown in previous studies, limited research has systematically reviewed the effects of EBN extract on cognitive function and neuroprotection in animal models. Therefore, the aims of the present paper are to address the following research questions: Does EBN extract significantly improve cognitive functions, such as memory and learning abilities, in animal models? Can EBN extract achieve neuroprotection by reducing oxidative stress or inflammation? Is there an optimal dosage range for EBN extract in cognitive enhancement? Can EBN extract mitigate cognitive decline in animal models of neurodegenerative diseases? Does EBN extract promote the expression of neurotrophic factors in the brain, thereby enhancing neuroplasticity?

## 2. Method

### 2.1. Data Collection and Processing

#### 2.1.1. Literature Search

Searches were conducted using PubMed, Scopus, Web of Science, EMBASE, Taylor Francis, Wiley, and Cochrane Library to identify original studies published up to October 2025. The search terms used were “Edible Bird’s Nest”, “Bird’s Nest Extract”, “EBN”, “Swiftlet nest”, “Collocalia”, “Cognitive”, “Memory”, “Learning”, “Neuroprotection”, “Brain”, “Neural”, “Neurotrophic”, “Animal”, “Mice”, “Mouse”, “Rat”, “Rats”, “In vivo”, and “Model”. Additional references for possible inclusion were obtained by manually scanning the reference lists of retrieved articles and systematic reviews.

The time frame of 2015–2025 was specifically selected based on advancements in EBN research methodologies in neuroprotection and cognitive function during this period and improvements in bioactive component analysis techniques for EBN, and our focus was to evaluate the most current scientific evidence. Although a comprehensive systematic review protocol was developed prior to conducting this review, including predefined research questions, search strategies, inclusion and exclusion criteria, and analysis methods, it was not registered in PROSPERO or other systematic review protocol registry centers.

#### 2.1.2. Search Statement

A systematic search strategy was developed to identify relevant studies across multiple databases. The search protocol combined specific terms related to edible bird’s nest with keywords associated with neuroprotection and cognitive function using Boolean operators (AND, OR). The detailed search terms and procedures for each database are presented in Table 1.

#### 2.1.3. Inclusion and Exclusion Criteria

Inclusion Criteria: ① animal studies using EBN as an intervention variable; ② studies focusing on cognitive function or neuroprotection-related fields; ③ experimental models must be animal models; ④ studies published between 2015 and 2025; ⑤ English-language publications; ⑥ studies that provide complete experimental data and results.

Exclusion Criteria: ① studies that do not use EBN as an independent intervention variable; ② non-animal studies (human studies or cell-based experiments); ③ studies not related to cognitive function or neuroprotection; ④ literature types such as reviews, commentaries, or meta-analyses; ⑤ studies written in languages other than English; ⑥ studies that do not provide complete experimental data.

#### 2.1.4. Paper Screening Results

**Figure 1:**
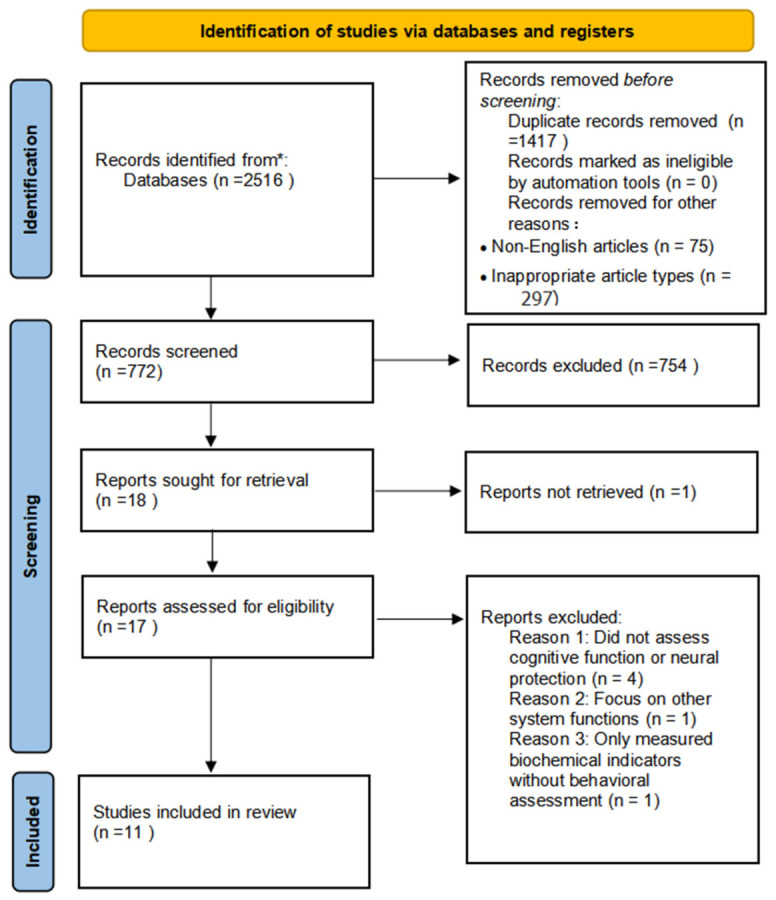
PRISMA flow diagram of the literature selection process. Note: * Records identified from databases include: Pubmed (n = 309), Scopus (n = 890), Web of Science (n = 549), Embase (n = 582), Taylor & Francis (n = 29), Wiley (n = 111), and Cochrane Library (n = 91).

A total of 2516 articles were initially identified from various databases: Pubmed (n = 309), Scopus (n = 890), Web of Science (n = 549), Embase (n = 582), Taylor & Francis (n = 29), Wiley (n = 111), and Cochrane Library (n = 91). After removing 1417 duplicate studies, 75 non-English publications, and 297 articles with incompatible types, 772 articles remained. Following title and abstract screening, 754 irrelevant or less relevant articles were excluded. The remaining 18 articles underwent full-text review, after which studies with irretrievable key data or high risk of bias were excluded, resulting in 17 articles. Based on the inclusion and exclusion criteria, 11 articles were ultimately selected for the final analysis. The systematic review was conducted in accordance with the Preferred Reporting Items for Systematic Reviews and Meta-Analyses (PRISMA) guidelines [23]. The systematic screening process is illustrated in Figure 1.

### 2.2. Data Extraction

Two researchers independently reviewed the titles and abstracts of 772 records. For inconsistencies in screening results, the researchers reached consensus through discussion to determine which articles required full-text review. When necessary, supervisors were consulted for final decisions. Subsequently, these two researchers independently evaluated the screened full-text articles to determine whether they met the inclusion criteria. Similarly, in cases of disagreement, consensus regarding inclusion or exclusion was reached through discussion.

### 2.3. Risk Assessment

**Figure 2:**
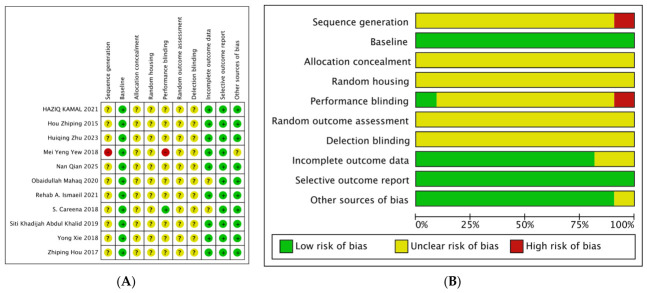
Bias risk assessment of included studies [25,26,27,28,29,30,31,32,33,34,35]. Note: (**A**) summary of bias risk; (**B**) bias risk graph. Legend: green (+) = low risk of bias; yellow (?) = unclear risk of bias; red (−) = high risk of bias.

This study employed the Systematic Review Centre for Laboratory Animal Experimentation (SYRCLE) risk of bias tool to evaluate the quality of the included literature [24]. The tool comprises 10 items across 6 dimensions—selection bias, performance bias, detection bias, attrition bias, reporting bias, and other biases—with risk levels categorized as “high risk,” “low risk,” or “unclear.” The assessment was independently conducted by two researchers, with any disagreements resolved by the supervisor to ensure objectivity and reliability of the evaluation results. The results are shown in Figure 2.

#### Integration of SYRCLE Risk Assessment Results

As shown in Figure 2, the SYRCLE risk assessment revealed several methodological concerns across the included studies. Selection bias was prominent, particularly in sequence generation and baseline characteristics, where most studies showed unclear risk. Performance bias was also notable in areas of random housing and blinding. These limitations were considered when interpreting the strength of evidence for each outcome domain, with findings from studies with higher risk of bias interpreted more cautiously.

### 2.4. Included Literature Review

A total of 11 English-language studies were included, primarily from research conducted in Asia. The experimental animals used in these studies mainly comprised C57BL/6J mice, SD rats, Wistar rats, ICR mice, and BALB/c mice, with sample sizes ranging from 3–4 per group to 42 animals. The research models covered various neurological disease or dysfunction models, including a Parkinson’s disease model (6-OHDA), ovariectomy-induced cognitive impairment model (OVX), brain inflammation model (LPS), cerebral ischemia model (2VO), and normal animal models. All interventions involved EBN administration, with dosages ranging from 10 mg/kg to 1.2 g/kg/day and intervention periods spanning from 21 days to 12 weeks. The main evaluation indicators included behavioral tests (Morris water maze, Y-maze, etc.) and molecular biological indicators (inflammatory factors, oxidative stress markers, gene expression, etc.). Results showed that EBN intervention demonstrated positive effects across various neurological disease models, including improved cognitive function, anti-inflammatory and antioxidant properties, and neuroprotective effects (Table 2).

## 3. Results

### 3.1. Improvement Effects of EBN Extract on Cognitive Functions in Animal Models

**Figure 3:**
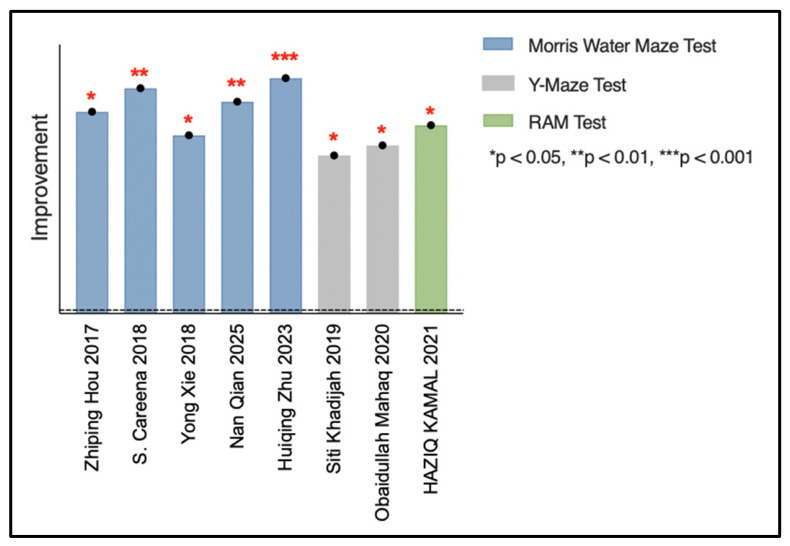
Comparative effects of Edible Bird’s Nest extract on cognitive function improvement in animal models [26,27,28,30,31,32,33,35].

EBN extract appears to improve cognitive function in several animal models, as shown in Figure 3. Morris water maze testing revealed significant enhancements in spatial learning and memory following EBN administration [26,27,28,32,33]. Animals treated with EBN demonstrated reduced escape latency and increased time in target quadrants, indicating better spatial navigation abilities.

Additional cognitive assessments further supported these findings. Y-maze testing showed enhanced spatial working memory [30,35], while radial arm maze assessment demonstrated improvements in both working and reference memory [31].

The cognitive benefits of EBN were evident across diverse experimental contexts: normal aging [31], ovariectomy-induced cognitive impairment [26], neuroinflammation models [27,32], and maternal–offspring transmission studies [28,33,35]. This consistency across varied models strengthens the evidence for EBN’s cognitive-enhancing properties.

Despite these promising results, critical evaluation reveals methodological limitations. The limited number of studies (only eight examining cognitive effects) restricts the strength of conclusions that can be drawn. Most studies utilized relatively small sample sizes, potentially limiting statistical power. Additionally, the heterogeneity in EBN preparation methods, dosing protocols, and cognitive assessment time frames creates substantial challenges for direct comparison between studies. This heterogeneity, combined with the limited study pool, makes it difficult to establish definitive dose–response relationships or determine which cognitive domains are most responsive to EBN intervention.

### 3.2. Antioxidant and Anti-Inflammatory Neuroprotective Mechanisms of EBN Extract

**Figure 4:**
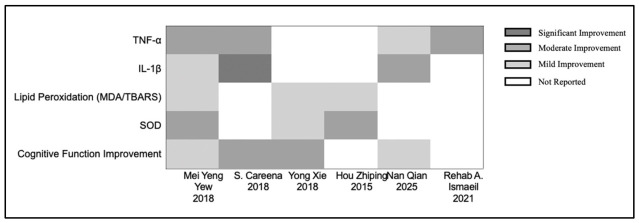
Comparative analysis of antioxidant and anti-inflammatory effects of Edible Bird’s Nest extract [25,27,28,29,32,34].

Available preclinical evidence suggests that EBN extract may possess antioxidant and anti-inflammatory properties across several disease models, as shown in Figure 4. Significant reductions in inflammatory mediators, including TNF-α and IL-1β, were observed following EBN treatment [25,32]. Molecular analysis revealed that EBN attenuates neuroinflammatory damage by inhibiting the NF-κB signaling pathway [32].

The antioxidant effects of EBN are well-documented, with evidence showing enhanced endogenous antioxidant defense systems. Multiple investigations confirmed increased antioxidant enzyme activities (SOD, GPx) and reduced lipid peroxidation markers (MDA/TBARS) [25,28,29]. EBN also inhibits nitric oxide production, providing specific protection to vulnerable dopaminergic neurons in Parkinson’s disease models [25].

Histological and functional assessments confirmed these neuroprotective effects. EBN treatment significantly reduced hippocampal neurodegeneration [29] and decreased F2-isoprostanes with corresponding amelioration of hippocampal damage in cerebral hypoperfusion models [34].

Critical analysis of these findings reveals several important limitations. The relatively small number of studies examining anti-inflammatory and antioxidant mechanisms (only six) limits the robustness of conclusions. The precise bioactive compounds responsible for these effects remain inadequately characterized, with most studies examining whole-extract effects rather than isolated components. Furthermore, the substantial heterogeneity in inflammatory markers measured across studies complicates direct comparisons and meta-analysis. The variability in disease models, from neuroinflammation to Parkinson’s disease to cerebral hypoperfusion, while demonstrating broad applicability, also introduces significant heterogeneity that challenges the development of a unified mechanistic understanding.

### 3.3. Dosage–Response Relationship of EBN Extract in Cognitive Function Studies

**Figure 5:**
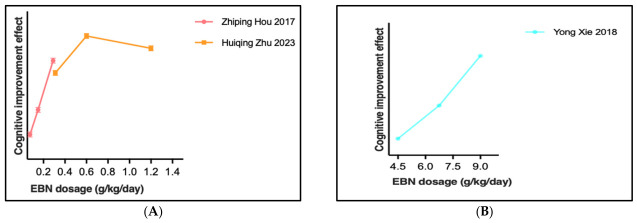
Effects of Edible Bird’s Nest extract dosage on cognitive function improvement [26,28,33]. Note: (**A**) low-dose Edible Bird’s Nest extract group; (**B**) high-dose Edible Bird’s Nest extract group.

Evidence indicates dose-dependent effects of EBN extract on cognitive enhancement, as shown in Figure 5. In ovariectomized rats, 1.2 g/kg/day showed superior efficacy compared to lower doses (0.3 and 0.6 g/kg/day), correlating with elevated hippocampal SIRT1 expression [26].

In maternal–offspring transmission models, maternal administration at 9.0 g/kg/day outperformed lower dosages (4.5 and 6.75 g/kg/day) in enhancing offspring cognitive capabilities [28]. A potential ceiling effect was observed at 1.5 g/kg/day through ERK-CREB-BDNF pathway activation, with higher dosages (3.0 g/kg/day) showing diminished benefits [33].

The preclinical evidence shows substantial dosage variability ranging from 1.2 to 9.0 g/kg/day across different experimental models. This wide range reflects heterogeneity in study designs and models rather than a defined therapeutic window. Given the eight-fold difference in dosages and variations in preparation methods, these findings should be interpreted cautiously, and further standardization is needed before establishing clinical dosing recommendations.

From a critical perspective, the wide variation in optimal dosages (1.2–9.0 g/kg/day) across studies raises concerns about translational applicability. This eight-fold difference in effective dosing suggests model-specific effects that may not generalize across conditions. Additionally, most studies employed relatively short treatment durations, leaving questions about long-term dosing requirements and potential tolerance effects unanswered. The lack of pharmacokinetic data for EBN components represents another significant gap, as absorption, distribution, and metabolism parameters remain largely unknown. These limitations underscore the preliminary nature of current dosing recommendations and highlight the need for more rigorous pharmacological characterization.

### 3.4. Neuroprotective Effects of EBN Extract on Cognitive Function in Animal Models of Neurodegenerative Diseases

**Figure 6:**
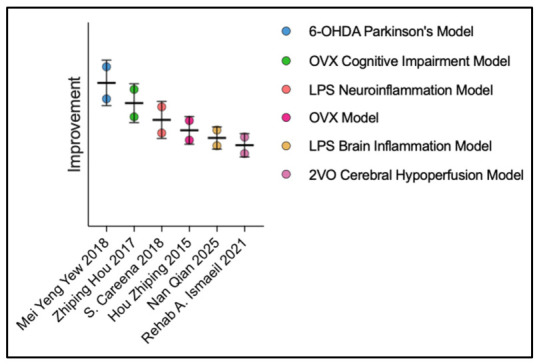
Comparative effects of Edible Bird’s Nest extract on cognitive decline improvement in various neurodegenerative disease models [25,26,27,29,32,34].

EBN extract demonstrates consistent neuroprotective efficacy across diverse neurodegenerative disease models, as shown in Figure 6. In ovariectomized (OVX) models simulating menopause-related cognitive decline, EBN administration mitigated cognitive impairment through increased hippocampal SIRT1 expression [26] and enhanced oxidative stress status [32].

In Parkinson’s disease models, EBN enhanced motor function and protected dopaminergic neurons against 6-OHDA-induced degeneration. The reduction in microglial activation and preservation of TH-positive neurons suggest that EBN addresses fundamental pathological processes in Parkinson’s disease [25].

In neuroinflammatory models, EBN improved LPS-induced learning and memory deficits by inhibiting inflammatory factor release (IL-6, TNF-α) and downregulating TLR4 expression [27,34]. Additionally, EBN attenuated neurodegenerative changes in cerebral hypoperfusion models, suggesting potential applications in vascular dementia [29].

The observed neuroprotective effects across these diverse pathological models raise the possibility that EBN might influence certain mechanisms common to multiple neurodegenerative processes, though this requires further investigation.

Critical evaluation of these findings reveals important translational limitations. The animal models employed, while valuable, represent simplified versions of complex human neurodegenerative conditions. Notably absent are studies using transgenic models of Alzheimer’s disease, which would provide more direct relevance to the most common form of dementia. Furthermore, most studies initiated EBN treatment simultaneously with or shortly after disease induction, representing a preventive rather than therapeutic approach. This timing does not reflect clinical reality, where treatment typically begins after substantial pathology has developed. The lack of studies comparing EBN efficacy to established pharmaceutical interventions also limits contextual understanding of its potential therapeutic value relative to current standards of care.

### 3.5. Mechanistic Pathways Potentially Underlying the Neuroplasticity-Enhancing Effects of EBN

**Figure 7:**
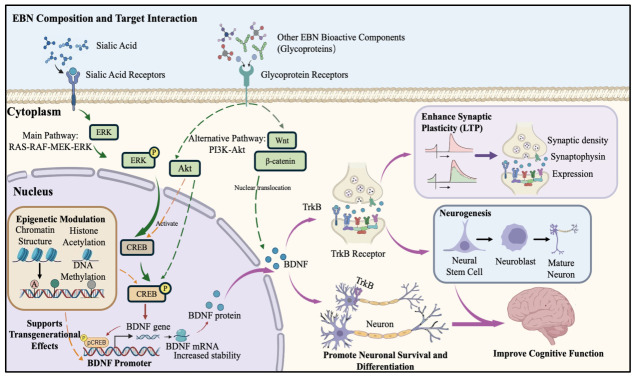
Molecular mechanisms of Edible Bird’s Nest in enhancing neuroplasticity [33]. (Note: Solid arrows indicate direct effects supported by experimental evidence, and dashed arrows represent alternative or indirect pathways. Orange arrows indicate epigenetic regulation, and purple arrows indicate BDNF-TrkB signaling).

Preclinical evidence suggests that the neuroplasticity-enhancing effects of EBN may be mediated through neurotrophic factor expression, particularly involving the ERK-CREB-BDNF signaling pathway, as shown in Figure 7. Several studies indicate that EBN administration is associated with neuroplasticity changes, with observed upregulation of BDNF protein and mRNA expression levels potentially contributing to the cognitive improvements noted in animal models [33]. The upregulated BDNF is thought to bind to its cognate receptor, tropomyosin receptor kinase B (TrkB), thereby activating downstream signaling cascades that promote synaptic plasticity, neurogenesis, and neuronal survival.

Some evidence suggests increased BDNF expression specifically in the hippocampal region of EBN-treated mice, accompanied by elevated neuronal counts [28]. This correlation between BDNF upregulation and increased neuronal density raises the possibility that EBN might support neurogenesis or neuronal survival, potentially through BDNF-TrkB receptor activation, though causal relationships remain to be established.

Preliminary transgenerational studies indicate that EBN supplementation may be associated with BDNF expression across multiple generations, alongside synaptic density and enhanced synaptic plasticity [35]. These observations hint at potential epigenetic mechanisms that might benefit cognitive function across generations, though this requires substantial further validation.

Current evidence suggests that sialic acid, a major component of EBN, could play a role in these neuroplasticity-enhancing effects, though other bioactive components likely contribute through complementary mechanisms that require further characterization.

From a critical perspective, the mechanistic studies exhibit several important limitations. While the ERK-CREB-BDNF pathway has been implicated, the upstream molecular targets through which EBN components initially engage with cellular machinery remain poorly defined. Additionally, the specific role of TrkB receptor activation in mediating EBN’s effects has not been directly investigated. The reliance on correlative rather than causative evidence—particularly regarding BDNF upregulation and TrkB activation in relation to cognitive outcomes—represents a significant methodological weakness. Inhibitor studies targeting TrkB or its downstream effectors, or genetic knockdown approaches would provide more definitive evidence for the necessity of these pathways in mediating EBN effects. Additionally, the transgenerational effects, while intriguing, require more rigorous epigenetic characterization to establish the specific modifications involved and their stability across generations. The focus on a limited set of molecular pathways may also overlook alternative mechanisms contributing to EBN’s neuroplasticity effects.

## 4. Discussion

This systematic review comprehensively analyzes 11 animal studies investigating the effects of EBN on cognitive function and neuroprotection. Available evidence indicates that EBN appears to show neuroprotective and cognitive-enhancing properties in animal models, with observed improvements in learning and memory abilities, particularly in spatial memory tasks. However, these findings are based on heterogeneous preclinical studies with methodological limitations. At the molecular level, EBN functions by enhancing antioxidant capacity, reducing neuroinflammation, promoting neurotrophic factor expression, and improving synaptic plasticity. These findings align with EBN’s characteristic richness in bioactive components such as sialic acid, providing scientific evidence for its potential as a neuroprotective agent.

Preclinical studies suggest that EBN’s potential neuroprotective effects may be achieved through multiple complementary mechanisms, though further research is needed to establish causal relationships. First, the high content of sialic acid (approximately 11%) in EBN plays a key role in promoting neuronal development and synaptic transmission [36]. Second, EBN’s antioxidant properties help neutralize free radicals and reduce oxidative stress, which is a major trigger for neurodegenerative diseases [37]. Third, EBN demonstrates significant anti-inflammatory effects by regulating inflammatory pathways, preventing chronic inflammation that leads to neuronal damage and cognitive impairment [38]. Additionally, EBN promotes the expression of neurotrophic factors, which are crucial for neuronal survival, differentiation, and synaptic plasticity [39].

Compared to plant extracts containing single active ingredients (such as ginkgo biloba or curcumin), EBN may provide more comprehensive neuroprotective effects [7].

This synergistic effect of multiple components enables EBN to simultaneously influence various neuroprotective pathways, including antioxidant [10], anti-inflammatory [40], and neurotrophic pathways [41], thereby forming a unique multi-target protective mechanism.

The current research field lacks experimental designs that directly compare the relative efficacy of EBN with other neuroprotective agents. Future research should focus on conducting controlled trials to systematically evaluate the effects of EBN used alone or in combination with other neuroprotective agents [42], determining its optimal positioning and application scenarios in neuroprotective strategies.

As a high-value product, establishing standardized production processes and quality assessment systems for EBN is key to promoting its widespread application. Innovative formulation technologies can improve the stability and bioavailability of active components in EBN [43].

From economic and ethical perspectives, developing sustainable bird’s nest farming methods [44], optimizing extraction processes to increase yield, and exploring alternative production methods may be ways to address this challenge [8], helping to translate bird’s nest research findings into neuroprotective products that benefit a wider population [1].

Despite encouraging results, there remain methodological limitations that need to be considered. First, the heterogeneity in bird’s nest preparation methods, dosages, and administration regimens makes direct comparison between studies complex [45]. Standardized procedures for bird’s nest extraction and characterization would facilitate more reliable comparison and analysis. Second, while animal models provide valuable insights for bird’s nest research, animal experiments may not fully reproduce the complexity of human neurodegenerative diseases or cognitive processes [46]. Translating these findings to human applications requires careful consideration of species differences in neurophysiology and metabolism.

This systematic review provides preliminary evidence for the cognitive enhancement and neuroprotective effects of EBN in animal models. Given the methodological limitations and heterogeneity across studies, EBN may warrant further investigation as a multifaceted neuroprotective agent that may be applied to the prevention and management of cognitive decline and neurodegenerative diseases. With further scientific validation and clinical research, EBN has the potential to become an effective complementary approach in combating neurodegenerative diseases.

Additionally, we acknowledge the methodological limitation that our systematic review protocol was not registered in advance. Protocol registration is an important step to enhance transparency and reduce reporting bias in systematic reviews. This limitation should be considered when interpreting our findings, although we strictly adhered to our predetermined methodology throughout the review process.

The SYRCLE risk assessment revealed significant methodological concerns that may affect the reliability of reported outcomes. The high prevalence of unclear reporting for randomization procedures, blinding, and allocation concealment represents a substantial limitation. These methodological weaknesses may lead to overestimation of treatment effects. The possibility of publication bias should also be acknowledged; as EBN is a high-value traditional product with commercial and cultural interests, we note that all included studies reported positive effects of EBN, and this consistency itself may reflect publication bias. These limitations do not completely negate the value of existing research, but they reveal that the results should be interpreted cautiously. Particularly when inferring optimal dosage ranges and cross-species translational applications, these methodological quality issues need to be considered. Future research should employ more rigorous experimental designs, including appropriate randomization, blinding, and sample size calculations, and should pre-register study protocols to reduce selective reporting bias.

Furthermore, this review is limited by the relatively small number of available studies on EBN’s neurological effects. The substantial heterogeneity across included studies presents additional challenges for interpretation. This heterogeneity manifests in several ways: (1) diverse animal models ranging from ovariectomized to LPS-induced to Parkinson’s models; (2) variable intervention protocols with EBN dosages varying from 0.3 to 9.0 g/kg/day; and (3) inconsistent outcome measurements across studies. The variability in EBN preparation methods is particularly noteworthy, as different extraction processes may yield products with varying bioactive component profiles.

While preclinical evidence suggests promising effects of EBN on neuroplasticity and cognition in animal models, several important translational limitations must be acknowledged. Species differences between rodents and humans in metabolism and neural systems necessitate caution when extrapolating these findings. The wide dosage range used in animal studies requires careful recalculation and safety assessment for human applications. Additionally, heterogeneity in EBN preparation methods across studies presents standardization challenges for clinical use. The relatively short intervention periods provide limited insight into long-term safety and efficacy profiles necessary for human therapeutic applications. These limitations highlight the preliminary nature of current evidence and underscore the substantial research required before EBN could be considered for human clinical applications.

## 5. Conclusions

This systematic review presents preliminary evidence regarding the neuroprotective and cognitive-enhancing properties of EBN in animal models. The identified mechanisms, including upregulation of neurotrophic factors and antioxidant effects, offer potential explanations for these observed outcomes. While findings show some consistency across diverse experimental models, the methodological limitations and heterogeneity in study designs necessitate cautious interpretation. Future research should focus on standardization and rigorous translational studies before clinical applications can be considered. With further validation through well-designed preclinical and clinical investigations, EBN may warrant consideration as a potential natural intervention for cognitive health and neuroprotection.

## Figures and Tables

**Table 1:** Search strategy and procedure in databases.

Database	Search Terms Protocol	Initial Result	Additional Information
PubMed			
1	(“Edible Bird’s Nest” OR “Bird’s Nest Extract” OR “EBN” OR “Swiftlet nest” OR “Collocalia”)	267	-Search conducted in the following fields: abstract and title. -Search conducted for 2015–2025 publication dates.
2	(“Edible Bird’s Nest” OR “Bird’s Nest Extract” OR “EBN” OR “Swiftlet nest” OR “Collocalia”) AND (“Cognitive” OR “Memory” OR “Learning” OR “Neuroprotection” OR “Brain” OR “Neural” OR “Neurotrophic”)	29	
3	(“Edible Bird’s Nest” OR “Bird’s Nest Extract” OR “EBN” OR “Swiftlet nest” OR “Collocalia”) AND (“Cognitive” OR “Memory” OR “Learning” OR “Neuroprotection” OR “Brain” OR “Neural” OR “Neurotrophic”) AND (“Animal” OR “Mice” OR “Mouse” OR “Rat” OR “Rats” OR “In vivo” OR “Model”)	13	
Scopus			
1	(“Edible Bird’s Nest” OR “Bird’s Nest Extract” OR “EBN” OR “Swiftlet nest” OR “Collocalia”)	760	-Search conducted in the following fields: article title, abstract and keywords. -Search conducted for 2015–2025 publication dates.
2	(“Edible Bird’s Nest” OR “Bird’s Nest Extract” OR “EBN” OR “Swiftlet nest” OR “Collocalia”) AND (“Cognitive” OR “Memory” OR “Learning” OR “Neuroprotection” OR “Brain” OR “Neural” OR “Neurotrophic”)	81	
3	(“Edible Bird’s Nest” OR “Bird’s Nest Extract” OR “EBN” OR “Swiftlet nest” OR “Collocalia”) AND (“Cognitive” OR “Memory” OR “Learning” OR “Neuroprotection” OR “Brain” OR “Neural” OR “Neurotrophic”) AND (“Animal” OR “Mice” OR “Mouse” OR “Rat” OR “Rats” OR “In vivo” OR “Model”)	49	
Web of Science			
1	(“Edible Bird’s Nest” OR “Bird’s Nest Extract” OR “EBN” OR “Swiftlet nest” OR “Collocalia”)	484	-Search conducted in the following fields: abstract. -Search conducted for 1 January 2015–1 October 2025 publication dates.
2	(“Edible Bird’s Nest” OR “Bird’s Nest Extract” OR “EBN” OR “Swiftlet nest” OR “Collocalia”) AND (“Cognitive” OR “Memory” OR “Learning” OR “Neuroprotection” OR “Brain” OR “Neural” OR “Neurotrophic”)	46	
3	(“Edible Bird’s Nest” OR “Bird’s Nest Extract” OR “EBN” OR “Swiftlet nest” OR “Collocalia”) AND (“Cognitive” OR “Memory” OR “Learning” OR “Neuroprotection” OR “Brain” OR “Neural” OR “Neurotrophic”) AND (“Animal” OR “Mice” OR “Mouse” OR “Rat” OR “Rats” OR “In vivo” OR “Model”)	19	
EMBASE			
1	(“Edible Birds Nest” OR “Birds Nest Extract” OR “EBN” OR “Swiftlet nest” OR “Collocalia”)	510	-Search conducted in the following fields: title and abstract. -Search conducted for 2015–2025 publication dates.
2	(“Edible Birds Nest” OR “Birds Nest Extract” OR “EBN” OR “Swiftlet nest” OR “Collocalia”) AND (“Cognitive” OR “Memory” OR “Learning” OR “Neuroprotection” OR “Brain” OR “Neural” OR “Neurotrophic”)	50	
3	(“Edible Birds Nest” OR “Birds Nest Extract” OR “EBN” OR “Swiftlet nest” OR “Collocalia”) AND (“Cognitive” OR “Memory” OR “Learning” OR “Neuroprotection” OR “Brain” OR “Neural” OR “Neurotrophic”) AND (“Animal” OR “Mice” OR “Mouse” OR “Rat” OR “Rats” OR “In vivo” OR “Model”)	22	
Taylor Francis			
1	(“Edible Bird’s Nest” OR “Bird’s Nest Extract” OR “EBN” OR “Swiftlet nest” OR “Collocalia”)	26	-Search conducted in the following fields: abstract. -Search conducted for 2015–2025 publication dates.
2	(“Edible Bird’s Nest” OR “Bird’s Nest Extract” OR “EBN” OR “Swiftlet nest” OR “Collocalia”) AND (“Cognitive” OR “Memory” OR “Learning” OR “Neuroprotection” OR “Brain” OR “Neural” OR “Neurotrophic”)	3	
3	(“Edible Bird’s Nest” OR “Bird’s Nest Extract” OR “EBN” OR “Swiftlet nest” OR “Collocalia”) AND (“Cognitive” OR “Memory” OR “Learning” OR “Neuroprotection” OR “Brain” OR “Neural” OR “Neurotrophic”) AND (“Animal” OR “Mice” OR “Mouse” OR “Rat” OR “Rats” OR “In vivo” OR “Model”)	0	
Wiley			
1	(“Edible Bird’s Nest” OR “Bird’s Nest Extract” OR “EBN” OR “Swiftlet nest” OR “Collocalia”)	97	-Search conducted in the following fields: abstract. -Search conducted for January 2015–October 2025 publication dates.
2	(“Edible Bird’s Nest” OR “Bird’s Nest Extract” OR “EBN” OR “Swiftlet nest” OR “Collocalia”) AND (“Cognitive” OR “Memory” OR “Learning” OR “Neuroprotection” OR “Brain” OR “Neural” OR “Neurotrophic”)	9	
3	(“Edible Bird’s Nest” OR “Bird’s Nest Extract” OR “EBN” OR “Swiftlet nest” OR “Collocalia”) AND (“Cognitive” OR “Memory” OR “Learning” OR “Neuroprotection” OR “Brain” OR “Neural” OR “Neurotrophic”) AND (“Animal” OR “Mice” OR “Mouse” OR “Rat” OR “Rats” OR “In vivo” OR “Model”)	5	
Cochrane Library			
1	(“Edible Bird’s Nest” OR “Bird’s Nest Extract” OR “EBN” OR “Swiftlet nest” OR “Collocalia”)	72	-Search conducted in the following fields: title, abstract and keywords -Search conducted for January 2015–October 2025 publication dates
2	(“Edible Bird’s Nest” OR “Bird’s Nest Extract” OR “EBN” OR “Swiftlet nest” OR “Collocalia”) AND (“Cognitive” OR “Memory” OR “Learning” OR “Neuroprotection” OR “Brain” OR “Neural” OR “Neurotrophic”)	15	
3	(“Edible Bird’s Nest” OR “Bird’s Nest Extract” OR “EBN” OR “Swiftlet nest” OR “Collocalia”) AND (“Cognitive” OR “Memory” OR “Learning” OR “Neuroprotection” OR “Brain” OR “Neural” OR “Neurotrophic”) AND (“Animal” OR “Mice” OR “Mouse” OR “Rat” OR “Rats” OR “In vivo” OR “Model”)	4	

**Table 2:** Basic information about the included literature.

First Author (Year)	Experimental Animals (Number)	Model Type	Intervention Protocol	Behavioral Tests	Main Evaluation Indicators	Results
Mei Yens Yew (2018) [25]	C57BL/6J mice (3–4 per cage)	6-OHDA Parkinson’s model	EBN: 20 and 100 mg/kg, 28 days	Locomotor activity tests (travel distance and balancing tests)	GPX1 expression, nitric oxide, CD11b, TH-positive neurons	Protection of dopaminergic neurons, improved locomotor activity, enhanced antioxidant capacity, reduced microglia activation, inhibited nitric oxide formation and lipid peroxidation
Zhiping Hou (2017) [26]	SD rats (42)	OVX cognitive impairment model	EBN: 0.3, 0.6, and 1.2 g/kg/day, 12 weeks	Morris water maze: 4 days of training (4 times/day) Day 5 exploration	Hippocampal SIRT1 expression	Improved spatial learning and memory, increased SIRT1 expression
S. Careena (2018) [27]	Wistar rats (male, 12–14 weeks, 250–300 g)	LPS neuroinflammation model	EBN: 125,500 mg/kg	Morris water maze: escape latency test and probe trial	Proinflammatory cytokines (TNF-α, IL-1β, IL-6), oxidative markers (ROS, TBARS) in hippocampus	Improved learning/memory, inhibited inflammation and oxidative stress
Yong Xie (2018) [28]	ICR mice (10 per group)	Pregnancy/lactation administration	Bird’s nest homogenate: 4.5 g/182 g (low), 6.75 g/182 g (middle), 9 g/182 g (high)	Morris water maze	MDA, SOD, ChAT, AChE, BDNF, neuron count in hippocampus	Improved offspring cognitive ability, increased SOD and ChAT activities, decreased MDA levels and AChE activities, enhanced BDNF levels and neuron counts in hippocampus
Hou Zhiping (2015) [29]	SD rats (42, female, 3-month-old, 180–200 g)	OVX model	1.5–6% bird’s nest feed, 12 weeks		AGEs, oxidative stress markers (MDA, SOD, CAT), neurodegeneration-related gene expression, Caspase 3 protein levels	Improved redox status, decreased AGEs, downregulated genes associated with neurodegeneration and apoptosis
Siti Khadijah Abdul Khalid (2019) [30]	BALB/c mice (female, 7-week-old)	Normal animals	Sialic acid 0.6 ppm, 21 days	Y-maze test	Y-maze performance (entries and time spent in novel arm)	Improved spatial memory
Haziq Kamal (2021) [31]	SD rats (24)	Normal animals	Traditional and Convenient EBN, 300 mg/kg, 42 days	RAM (8-arm maze): 3 days for shaping, 8 days for acquisition, 1 day for retention	RAM test performance (WME, RME, time spent, percentage of correct choices)	Improved working and spatial memory, TEBN better than CEBN
Nan Qian (2025) [32]	C57BL/6J mice (32, female, 7-week-old)	LPS brain inflammation model	EBN: 200 mg/kg/d (dry weight), compared with free SA	Morris water maze, Open-Field Test	Inflammatory factors (TNF-α, IL-1β, IL-6), immunohistochemistry (GFAP, IBA-1), NF-κB pathway markers	Improved cognitive function, reduced escape latency, anti-anxiety effects, inhibited neuroinflammation, alleviated hippocampal damage
Huiqing Zhu (2023) [33]	SD rat offspring (from 30 female and 15 male parents)	Prenatal nutrition intervention	EBN: 4.5–9 g/kg/d (maternal supplementation during pregnancy and lactation)	Morris water maze	ERK-CREB-BDNF pathway, synaptic density, hippocampal neurogenesis	Promoted hippocampal development, enhanced synaptic plasticity, improved learning and memory function
Rehab A. Ismaeil (2021) [34]	SD rats (24, male, 200–250 g)	2VO cerebral hypoperfusion model	EBN: 60–120 mg/kg, daily oral gavage, 8 weeks		F2-isoprostanes (ELISA), hippocampal CA1 neuron count (histopathology)	Reduced neuronal death in CA1 region, decreased oxidative stress markers (F2-isoprostanes)
Obaidullah Mahaq (2020) [35]	C57BL/6 mice (F0, F1, and F2 generations)	Normal animals	EBN: 10 mg/kg (administered to F0 breeders for 6 weeks)	Y-maze test	Gene expression (GNE, ST8SiaIV, SLC17A5, BDNF mRNA), synaptic vesicle density	Improved cognitive function in Y-maze test, increased synaptic density, upregulated genes related to sialic acid metabolism and neural plasticity

Note: EBN: Edible Bird’s Nest; TEBN: Traditional Edible Bird’s Nest; CEBN: Convenient Edible Bird’s Nest; SA: sialic acid; LPS: Lipopolysaccharide; OVX: ovariectomized; 6-OHDA: 6-hydroxydopamine; 2VO: Bilateral Common Carotid Artery Occlusion; RAM: radial arm maze; WME: working memory error; RME: reference memory error; TNF-α: Tumor Necrosis Factor-alpha; IL-1β: Interleukin-1 beta; IL-6: Interleukin-6; BDNF: Brain-Derived Neurotrophic Factor; GFAP: Glial Fibrillary Acidic Protein; IBA-1: ionized calcium-binding adapter molecule 1; NF-κB: Nuclear Factor-kappa B; ERK: Extracellular Signal-Regulated Kinase; CREB: cAMP Response Element-Binding Protein.

## Data Availability

The data supporting the findings of this systematic review are available within the article. All articles included in this review were obtained from publicly accessible databases including PubMed, Scopus, Web of Science, EMBASE, Taylor Francis, Wiley, and Cochrane Library. The search strategy, inclusion/exclusion criteria, quality assessment methods, and data extraction protocols are fully described in Section 2. The complete list of included studies, their characteristics, and the extracted data are presented in the tables and figures within the manuscript. The SYRCLE risk of bias assessment results for each included study are available in the tables and figures within the manuscript. No additional datasets were generated during this systematic review. Any reasonable requests for further information should be directed to the corresponding author.
